# Identification of pyroptosis and hypoxia related molecular subtypes and a prognostic signature in colorectal cancer

**DOI:** 10.1007/s12672-026-04970-w

**Published:** 2026-04-22

**Authors:** Hui Wang, YuanWang Chen

**Affiliations:** 1 Department of Critical Care Medicine, The Second Affiliated Hospital, Jiangxi Medical College, Nanchang University, Nanchang, Jiangxi China; 2 Jiujiang City Key Laboratory of Cell Therapy, The First People’s Hospital of Jiujiang City, Jiujiang, Jiangxi China

**Keywords:** Colorectal cancer, Hypoxia, Pyroptosis, Molecular subtypes, Prognostic signature, Immunotherapy

## Abstract

**Background:**

Colorectal cancer (CRC) is one of the most common malignancies of the gastrointestinal tract and remains a leading cause of cancer-related mortality. Hypoxia and pyroptosis are closely linked to malignant progression and may shape tumour biology and the immune microenvironment.

**Methods:**

Pyroptosis- and hypoxia-related genes were obtained from GeneCards and MSigDB, and PHRGs were defined as the intersection of pyroptosis- and hypoxia-associated gene sets for subsequent consensus clustering. Transcriptomic profiles and survival information for CRC were retrieved from The Cancer Genome Atlas (TCGA) and Gene Expression Omnibus (GEO) databases. Pyroptosis- and hypoxia-related molecular subtypes were identified using consensus clustering. Least absolute shrinkage and selection operator and Cox regression analyses were applied to construct a prognostic signature. Key signature genes were preliminarily assessed at the expression level in paired CRC and adjacent normal tissues using qRT-PCR (*n* = 10 pairs) and western blotting (*n* = 6 pairs). Given the limited sample size and lack of stage- or subtype-stratified validation, these experiments provide preliminary expression-level support rather than functional confirmation.

**Results:**

Patients were classified into four PHRG-related molecular subtypes with distinct prognoses and biological characteristics, with PHRGcluster C2 showing the worst outcome. A total of 1578 pyroptosis- and hypoxia-related differentially expressed genes (PHRDEGs) were identified across the four PHRGclusters. A six-gene prognostic signature (PHRDEGscore) was then established in the TCGA cohort. Patients with a high PHRDEGscore had shorter survival, which was validated in external cohorts (GSE39582 and GSE17536). The low-PHRDEGscore group exhibited higher immune infiltration (e.g., activated CD8+ and CD4+ T cells), and an exploratory association with immunotherapy response was observed in a non-CRC immunotherapy-treated cohort (IMvigor210). Experimental analyses indicated higher mRNA levels of HES4, SHROOM2, and OXCT1 in CRC tissues than in adjacent normal tissues (qRT-PCR, *n* = 10 pairs), and increased protein expression of HES4 and SHROOM2 (western blotting, *n* = 6 pairs).

**Conclusions:**

This study identified hypoxia- and pyroptosis-related molecular patterns and a six-gene prognostic signature in CRC. The observed associations are correlative and hypothesis-generating. Prospective validation in independent, well-annotated clinical cohorts is needed before any clinical application can be considered.

**Supplementary Information:**

The online version contains supplementary material available at 10.1007/s12672-026-04970-w.

## Introduction

Colorectal cancer (CRC) is one of the most common malignancies of the gastrointestinal system, causing nearly 903,900 deaths in 2022. According to the latest data released by the International Agency for Research on Cancer (IARC), the number of new CRC cases in 2022 was 1.92 million. It is the third most commonly diagnosed cancer in the world, after lung and breast cancer, and the second leading cause of cancer death (after lung cancer) [1, 2]. CRC develops through multiple factors, including genetics, family history, dietary habits, obesity, alcohol consumption, and smoking [3]. Approximately 10% of patients with CRC have hereditary CRC [4]. Recently, CRC incidence has shown a concerning shift toward younger populations, with increasing diagnoses in individuals under 50 years of age, posing a growing global health burden [5]. Studies have shown that the 5-year survival rate of patients with CRC is approximately 65% and that of patients with stage I, II, and IV CRC is 91%, 82%, and 12%, respectively [6, 7]. Radical surgery remains a cornerstone of CRC treatment and contributes to improved prognosis. Chemotherapy, radiotherapy, targeted therapy, and immunotherapy are important modalities for postoperative management and metastatic CRC, helping to reduce recurrence and prolong survival [8]. However, not all patients benefit from adjuvant therapy. Advanced or metastatic disease, drug resistance, and a complex tumour microenvironment remain major barriers to improved outcomes [9, 10]. Hence, identifying reliable diagnostic and therapeutic biomarkers is essential to refine risk stratification and improve CRC prognosis.

Hypoxia, a key feature of the tumour microenvironment, is closely linked to metabolic reprogramming, angiogenesis, immune regulation, and metastasis in malignant tumours [11–13]. Rapid proliferation of malignant cells can outpace vascular growth, leading to oxygen and nutrient scarcity within the tumour microenvironment. These constraints can drive genomic and proteomic alterations that influence tumour pathophysiology and prognosis [14–16]. Malignant tumour cells adapt to hypoxia largely through hypoxia-inducible factors (HIFs), which are dimers consisting of oxygen-sensitive α subunits (HIF-1α, HIF-2α, and HIF-3α) and a scaffold β subunit [17]. Krishnamachary et al. [18] reported that HIF-1 can activate genes involved in tumour angiogenesis and glycolytic metabolism, and that hypoxia and HIF-1α can increase the aggressiveness of colon carcinoma. Cao et al. [19] showed a significant positive correlation between HIF-1α and VEGF expression and reported that HIF-1α was strongly associated with the TNM stage of CRC. In addition, hypoxia plays a crucial role in treatment response, contributing to radiation resistance, chemotherapy resistance, and immunosuppression [20]. Nutrient and oxygen deprivation within the tumour microenvironment can intensify metabolic competition between tumour and immune cells. The robust metabolic capacity of tumour cells can impose metabolic stress on immune cells and promote the accumulation of toxic metabolites, thereby weakening antitumour immunity and facilitating immune escape [21].

Unlike traditional apoptosis and cell necrosis, pyroptosis is a distinct form of inflammatory programmed cell death that has been increasingly recognized in recent years [22]. Gasdermin family proteins are key mediators of pyroptosis and mainly include GSDMA/B/C/D and GSDME [23, 24]. Pyroptosis occurs through two major pathways: a classical pathway driven by caspase-1 activation and a non-classical pathway driven by caspase-4/5/11 activation. Inflammasome activation triggers caspase-1/4/5/11, leading to cleavage and activation of GSDMD. Activated GSDMD forms pores in the cell membrane, causing membrane rupture and release of pro-inflammatory contents, thereby eliciting a strong inflammatory response within the tumour microenvironment [22]. Pyroptosis appears to play dual roles in malignant tumours. On the one hand, certain therapeutics can induce tumour cell death by engaging pyroptotic pathways [25]. On the other hand, pyroptosis can amplify local inflammation and contribute to an immunosuppressive tumour microenvironment [26]. Accumulating evidence suggests that pyroptosis is associated with malignant tumour development, invasion, metastasis, and immunotherapy response [27–30].

We used pyroptosis- and hypoxia-related genes to characterize CRC molecular patterns and to explore their associations with pathway activity and the tumour microenvironment. In addition, we constructed a prognostic model derived from retrospective public cohorts and evaluated its relationships with immune features and immunotherapy response in available datasets, while acknowledging that mechanistic causality and prospective clinical utility require further validation.

## Materials and methods

### Data sources

Transcriptomic data and clinical information for colon adenocarcinoma (COAD) and rectal adenocarcinoma (READ) were obtained from The Cancer Genome Atlas (TCGA, https://portal.gdc.cancer.gov/) using the TCGAbiolinks package (Table 1) [31]. Given the shared biological and clinical characteristics of colorectal cancer (CRC), COAD and READ datasets were combined, yielding a total of 460 CRC patients, including 401 censored (alive) and 59 deceased cases. Masked somatic mutation and masked copy number segment data were also downloaded from TCGA. Somatic mutation landscapes were visualized using the maftools package [32], and copy number variation (CNV) profiles were analyzed using ggplot2.CRC-related GEO datasets, GSE39582 (585 samples) [33] and GSE17536 (177 samples) [34], were downloaded from the Gene Expression Omnibus (GEO; https://www.ncbi.nlm.nih.gov/geo/) using the GEOquery package [35]. An immunotherapy-treated cohort for bladder cancer was accessed via the IMvigor210CoreBiologies package [36]. Pyroptosis-related genes were retrieved from the GeneCards database (https://www.genecards.org/) [37] using the keyword “pyroptosis”. To reduce non-specific associations and improve reproducibility, genes were filtered using a predefined GeneCards relevance score threshold (relevance score ≥ 10). Only protein-coding genes with official HGNC symbols were retained, resulting in 372 pyroptosis-related genes (Table S1). Hypoxia-related genes were obtained from the Molecular Signatures Database (MSigDB; https://www.gsea-msigdb.org/gsea/index.jsp) [38]. The curated HALLMARK_HYPOXIA gene set (200 genes; Table S2) was selected, as hallmark gene sets represent well-defined biological states with reduced redundancy and improved robustness across cohorts. Pyroptosis- and hypoxia-related genes (PHRGs) were defined as the intersection of the two gene sets, yielding 13 genes after symbol standardization and deduplication (Table S3). This intersection-based strategy was adopted to focus on genes jointly implicated in hypoxia and pyroptosis, thereby enhancing biological specificity and reducing noise in downstream clustering and prognostic modeling.

**Table 1:** TCGA dataset information list

	Alive (401)	Dead (59)	Total (460)
Age			
Mean	64.7	68.4	65.2
Median	66	71	67
≤67	217 (54%)	17 (29%)	234 (51%)
> 67	184 (46%)	42 (71%)	226 (49%)
Gender			
Female	182 (45%)	25 (42%)	207 (45%)
Male	219 (55%)	34 (58%)	253 (55%)
T			
T1	14 (3.5%)	2 (3.4%)	16 (3.5%)
T2	72 (18%)	4 (6.8%)	76 (16.5%)
T3	275 (68.6%)	40 (67.8%)	315 (68.5%)
T4	39 (9.7%)	12 (20.3%)	51 (11.1%)
TX	1 (0.2%)	1 (1.7%)	2 (0.4%)
N			
N0	234 (58.4%)	23 (39%)	257 (55.9%)
N1	9 9(24.7%)	18 (30.5%)	117 (25.4%)
N2	66 (16.4%)	17 (28.8%)	83 (18%)
NX	2 (0.5%)	1 (1.7%)	3 (0.7%)
M			
M0	298 (74.3%)	31 (52.5%)	329 (72%)
M1	45 (11.2%)	20 (33.9%)	65 (14%)
MX	58 (14.5%)	8 (13.6%)	66 (14%)
Stage			
I	73 (18.2%)	3 (5%)	76 (16.5%)
II	146 (36.4%)	17 (28.8%)	163 (35.4%)
III	120 (30%)	15 (25.4%)	135 (29.3%)
IV	46 (11.4%)	20 (34%)	66 (14.4%)
Site			
Colon	290 (72%)	50 (85%)	340 (74%)
Rectum	111 (28%)	9 (15%)	120 (26%)

*TCGA* The Cancer Genome Atlas

### Construction of molecular subtypes based on pyroptosis- and hypoxia-related genes

To identify CRC molecular subtypes, consensus clustering analysis was performed using the ConsensusClusterPlus package [39] based on the expression profiles of PHRGs in the TCGA cohort. The number of clusters was evaluated from 2 to 8, with 1000 resampling iterations, each including 80% of the samples. Partitioning around medoids (clusterAlg = “pam”) and Euclidean distance were applied. Gene set variation analysis (GSVA) was conducted to estimate pathway activity at the individual-sample level using the GSVA package with default parameters. Hallmark gene sets (h.all.v7.5.1.symbols) from MSigDB were used as reference pathways. Differences in GSVA enrichment scores among molecular subtypes were assessed within the limma framework, and multiple testing was corrected using the Benjamini–Hochberg method. Pathways with an adjusted P value (FDR) < 0.05 were considered statistically significant.

### Analysis of differential genes associated with pyroptosis and hypoxia

To further evaluate the impact of PHRDEGs on CRC survival, consensus clustering analysis was again performed using the ConsensusClusterPlus package [39] based on PHRDEG expression profiles. The number of clusters was set between 2 and 8, with 1000 resampling iterations including 80% of samples per iteration, using the pam algorithm and Euclidean distance. Survival differences among the resulting clusters were subsequently compared.

### Construction of molecular subtypes based on pyroptosis- and hypoxia-related differentially expressed genes

To further determine what effect the PHRDEGs have on CRC survival time, the ‘ConsensusClusterPlus’ package [39] was used to conduct consistency clustering analysis for patients with CRC based on the PHRDEGs. In this method, the quantity of clusters was set between 2–8, replicated 1000 times to extract 80% of all samples, clusterAlg = ‘pam’, and distance = ‘euclidean’”. Survival times within various clusters were further compared.

### Enrichment analysis

Gene Ontology (GO) analysis was conducted to characterize the biological process (BP), molecular function (MF), and cellular component (CC) associated with the identified genes [40]. Kyoto Encyclopedia of Genes and Genomes (KEGG) pathway analysis was performed to investigate relevant biological pathways [41]. GO and KEGG enrichment analyses were conducted using the clusterProfiler package, with an FDR < 0.05 considered statistically significant.

### Construction of a prognostic signature using pyroptosis- and hypoxia-related differentially expressed genes

Least absolute shrinkage and selection operator (LASSO) regression [42] was applied to reduce overfitting by introducing penalty terms into the regression model. To evaluate the prognostic value of PHRDEGs, univariate Cox regression analysis was first performed to identify genes significantly associated with overall survival. LASSO regression was then used to eliminate multicollinearity and select candidate genes. Finally, multivariate Cox regression analysis was conducted to identify independent prognostic genes and construct the PHRDEGscore model.

The PHRDEGscore was calculated as follows:

$${\mathrm{PHRDEGscore}}=\sum {{\mathrm{Coefficient}}\left( {{\mathrm{gen}}{{\mathrm{e}}_{\mathrm{i}}}} \right) \times {\mathrm{mRNA}}\;{\mathrm{Expression}}\left( {{\mathrm{gen}}{{\mathrm{e}}_{\mathrm{i}}}} \right)} $$

The surv_cutpoint function was used to determine the optimal cutoff value of the PHRDEGscore, and patients were stratified into high- and low-risk groups accordingly. The GSE17536 and GSE39582 datasets were used for external validation of the prognostic performance of the model.

### Gene set enrichment analysis

Gene set enrichment analysis (GSEA) was performed to explore biological differences between high- and low-PHRDEGscore groups. The h.all.v7.5.1.symbols gene set was used as the reference. An adjusted P value < 0.05 was considered statistically significant.

### Immune infiltration analysis

Immune cell infiltration was evaluated using single-sample gene set enrichment analysis (ssGSEA). A previously published immune-related gene set comprising 782 genes representing 28 immune cell subsets was used as reference [43]. ssGSEA enrichment scores were calculated for each sample, and differences between groups (e.g., high vs. low PHRDEGscore) were assessed using two-sided Wilcoxon rank-sum tests. When multiple immune cell subsets were analyzed simultaneously, P values were adjusted using the Benjamini–Hochberg method, and an FDR < 0.05 was considered statistically significant.

### Establishment of a nomogram

A nomogram was constructed based on multivariate Cox regression analysis to estimate individual survival probabilities [44]. Univariate and multivariate Cox regression analyses were conducted to assess whether the PHRDEGscore combined with clinicopathological variables could predict patient survival. Variables significantly associated with prognosis were incorporated into the nomogram using the rms package. Calibration curves were generated to evaluate the predictive accuracy of the nomogram.

### In-vitro verification of signature genes using qRT-PCR

Ten pairs of CRC tissues and adjacent normal tissues were collected from patients who underwent surgical resection at The First People’s Hospital of Jiujiang City between July 2024 and September 2024. The study was approved by the Ethics Committee, and all participants provided written informed consent. Tissue samples were stored at − 80 °C until use. Total RNA was extracted using TRIzol reagent (Invitrogen), and cDNA was synthesized using the PrimeScript RT kit (Vazyme, Nanjing, China). Quantitative real-time PCR was performed using SYBR Premix Ex Taq (Takara Bio, Inc.) on an ABI 7500 Rapid Real-Time PCR System (Applied Biosystems; Thermo Fisher Scientific, Inc.). Relative gene expression levels were calculated using the 2^−ΔΔCt method with GAPDH as the internal reference. Differences in expression between paired tumor and adjacent normal tissues were assessed using paired t-tests or Wilcoxon signed-rank tests, as appropriate. Primer sequences are listed in Table 2. Given the limited sample size and lack of stratification by clinical stage or molecular subtype, this validation was intended as an exploratory expression-level assessment rather than definitive functional or clinical validation.

**Table 2:** Primer sequences for PCR

Names	Upstream sequence	Downstream sequence
GAPDH	TGACGTGGACATCCGCAAAG	CTGGAAGGTGGACAGCGAGG
TM4SF4	AGGAAGCGGTGTCTTGATGA	ACGAGTATCCAGCTCCCAAG
OXCT1	GCTTTGGTGAAAGCCTGGAA	CACTGTGGTTTCTGCAGCTT
HES4	CAAGCCGGTCATGGAGAAG	AGGTGTCTCACGGTCATCT
GAL	GTTGGCAACCACAGGTCATT	ACCTGTCAAAGCTTCCTGGT
HLA-DQB2	GCATCGAGGACTGGAACAAC	GTTGTGTCTGCACACCTTGT
SHROOM2	GGGCTGGAGAAAGACCAGAT	AGACAGAAGCGCGAGTAGAA

### Western blotting

Western blotting was performed to validate protein expression of differentially expressed genes identified by qRT-PCR. Paired CRC and adjacent normal tissue samples were homogenized in RIPA lysis buffer (R0010, Solarbio). Equal amounts of protein were separated by 10% SDS–PAGE and transferred onto polyvinylidene fluoride (PVDF) membranes. Membranes were blocked with 5% BSA and incubated overnight at 4 °C with primary antibodies against β-actin, HES4, SHROOM2, and OXCT1. After washing three times with TBST, membranes were incubated with the appropriate secondary antibodies for 1 h at room temperature. Protein bands were visualized using enhanced chemiluminescence and recorded using a gel imaging system. Relative protein expression levels were quantified using β-actin as the internal control.

The antibodies and dilutions used were as follows: anti-SHROOM2 (SAB2700199, 1:1000; Sigma), anti-OXCT1 (81011-1-RR, 1:5000; Proteintech), anti-HES4 (sc-376002, 1:500; Santa Cruz Biotechnology), and anti-β-actin (HC201, 1:4000; TransGen Biotech).

### Statistical analysis

For pathway activity estimation, GSVA enrichment scores were calculated at the sample level using the GSVA package with default parameters. Differences in GSVA scores between groups were evaluated using limma, with multiple testing corrected by the Benjamini–Hochberg method; pathways with an FDR < 0.05 were considered statistically significant. For immune infiltration analyses, ssGSEA scores were compared between groups using two-sided Wilcoxon rank-sum tests, and multiple comparisons were corrected using the Benjamini–Hochberg method. An FDR < 0.05 was considered statistically significant.

## Results

### Genetic alterations of pyroptosis- and hypoxia-related genes in The Cancer Genome Atlas-colorectal cancer

**Fig. 1:**
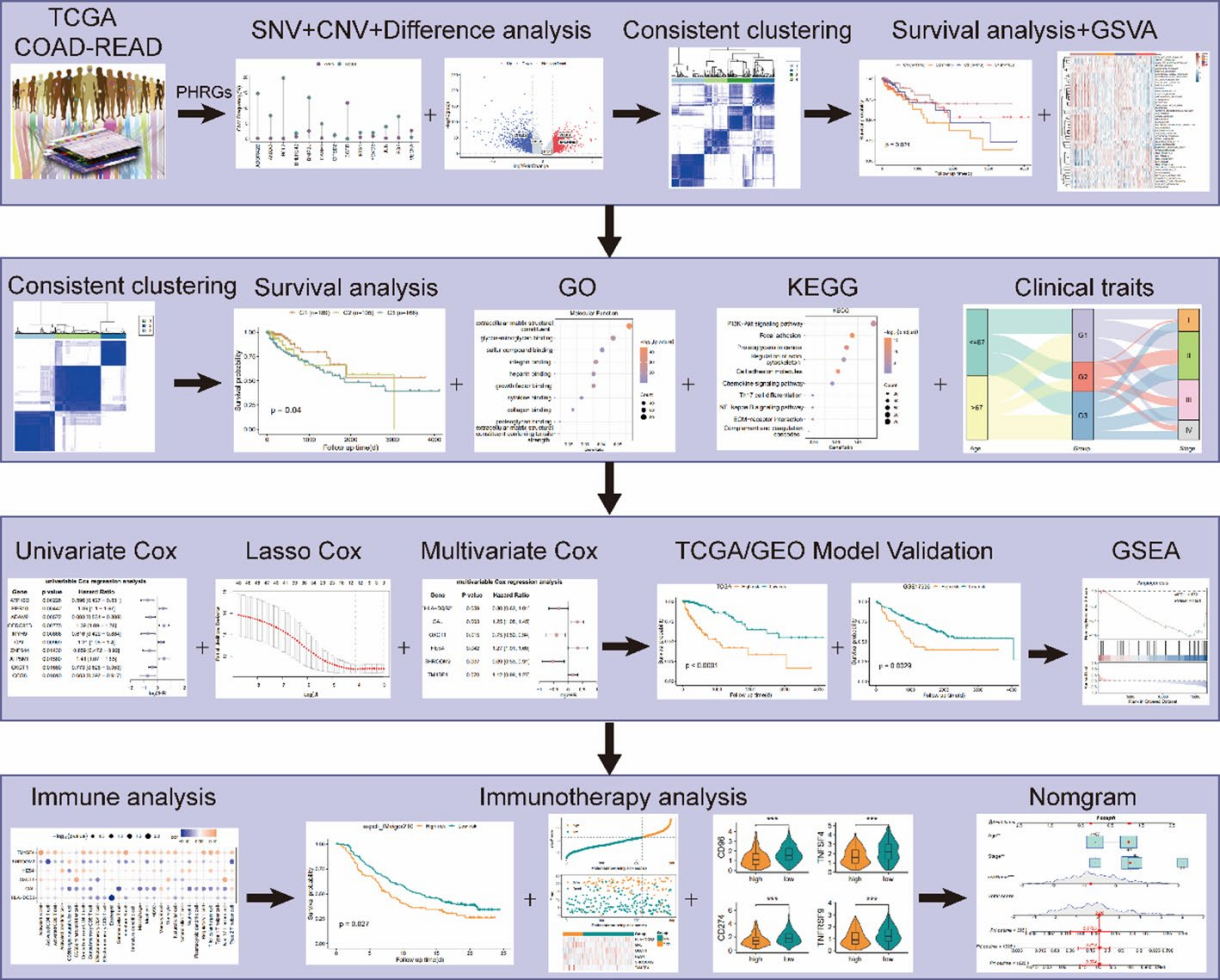
Flowchart of the overall study design and analytical workflow. The diagram summarizes data collection, identification of pyroptosis- and hypoxia-related genes (PHRGs), molecular subtype classification, prognostic model construction, immune and immunotherapy analyses, and experimental validation

**Fig. 2:**
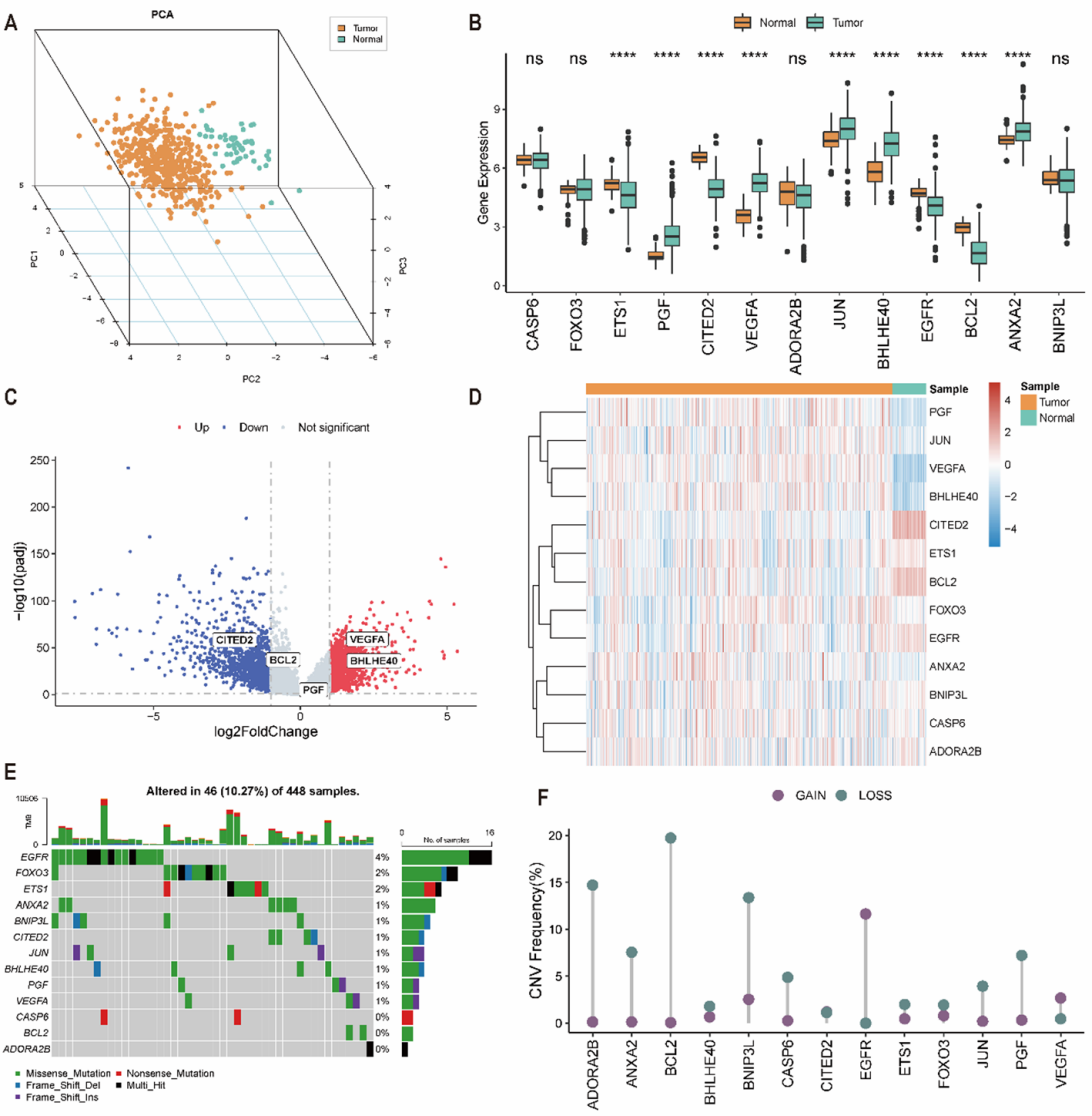
Landscape of pyroptosis- and hypoxia-related genes (PHRGs) in colorectal cancer (CRC). **A** Principal component analysis (PCA) based on PHRG expression profiles distinguishing CRC and adjacent normal tissues. **B** Differential expression of PHRGs between CRC and adjacent normal tissues. **C** Volcano plot and **D** heatmap illustrating PHRG expression differences. **E** Waterfall plot showing somatic mutation profiles of PHRGs in CRC. **F** Copy number variation (CNV) patterns of PHRGs across CRC samples

The overall workflow of this study is summarized in Fig. 1. To evaluate the potential relevance of pyroptosis- and hypoxia-related genes (PHRGs) in CRC, principal component analysis (PCA) was first performed on the TCGA-CRC cohort using the prcomp function based on the expression profiles of PHRGs. The PCA results indicated that PHRGs could largely distinguish tumour samples from normal samples (Fig. 2A). Examination of PHRG mRNA expression further showed that more than 60% of PHRGs were significantly differentially expressed between colorectal tumour and normal tissues (Fig. 2B). Differential expression was subsequently assessed using the limma package and visualized by a volcano plot (Fig. 2C) and heatmap (Fig. 2D). Specifically, ETS1, EGFR, BCL2, and CITED2 showed significantly lower expression in tumour tissues, whereas JUN, ANXA2, BHLHE40, PGF, and VEGFA were significantly upregulated. To characterize genomic alterations, somatic mutations of PHRGs were analyzed using the maftools package. PHRG mutations were observed in 46 samples, corresponding to a mutation frequency of 10.27% (Fig. 2E). Copy number variation (CNV) analysis showed that most PHRGs exhibited CNV events, with copy number loss being more prominent than gain (Fig. 2F).

### Construction of pyroptosis- and hypoxia-related gene-related molecular subtypes

**Fig. 3:**
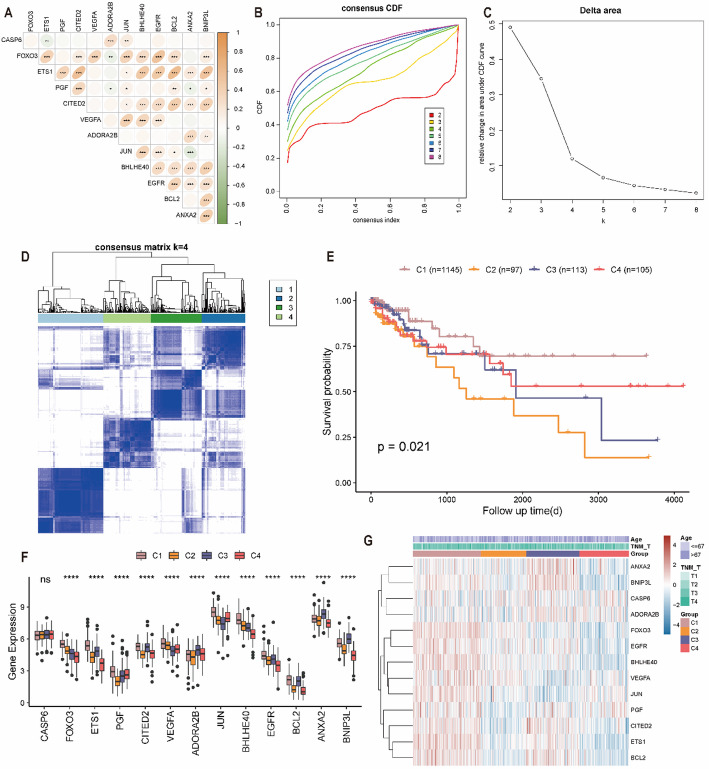
Identification of pyroptosis- and hypoxia-related molecular subtypes based on PHRGs. **A** Correlation matrix of PHRG expression. **B** Cumulative distribution function (CDF) curves for consensus clustering. **C** Relative change in the area under the CDF curve. **D** Heatmap of the consensus matrix at the optimal cluster number. **E** Kaplan–Meier survival curves of the four PHRG-based molecular subtypes (PHRGclusters). **F** Expression patterns of PHRGs across different PHRGclusters. **G** Heatmap showing differential PHRG expression among PHRGclusters

Correlation analysis showed that most PHRGs were positively correlated with each other, and ETS1 exhibited a strong positive correlation with BCL2 and CITED2 (Fig. 3A). Based on PHRG expression profiles, consensus clustering analysis stratified all patients into four PHRGclusters (Fig. 3B–D). Kaplan–Meier analysis demonstrated significant survival differences among the four PHRGclusters (Fig. 3E). The expression distribution of PHRGs across clusters was visualized using ggplot2, showing that, except for CASP6, PHRGs differed significantly among PHRGclusters (Fig. 3F). Consistently, the heatmap indicated that PGF, CITED2, and ETS1 were highly expressed in PHRGcluster C1 (favorable prognosis) but expressed at lower levels in PHRGcluster C2 (unfavorable prognosis) (Fig. 3G).

### Gene set variation analysis of pyroptosis- and hypoxia-related gene-related molecular subtypes

**Fig. 4:**
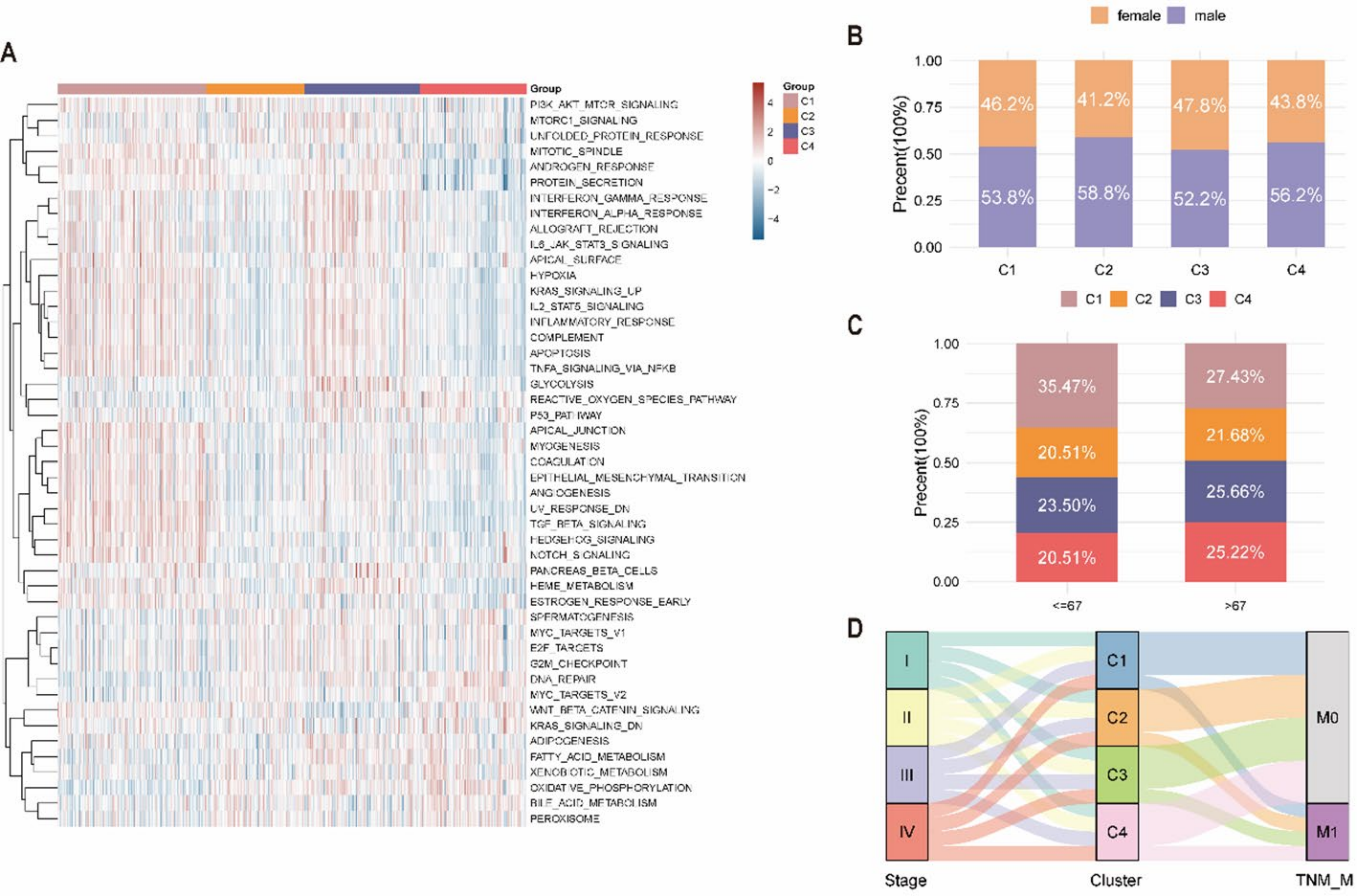
Functional characteristics and clinical associations of PHRG-related molecular subtypes. **A** Gene set variation analysis (GSVA) comparing pathway activities among PHRGclusters. **B** Distribution of sex across PHRGclusters. **C** Distribution of age across PHRG-related molecular subtypes. **D** Alluvial diagram illustrating relationships among tumour stage, PHRGclusters, and TNM_M status

To investigate functional differences among PHRGclusters, GSVA was performed (Fig. 4A, Table S4). Enriched pathways included HALLMARK_UV_RESPONSE_DN, HALLMARK_TGF_BETA_SIGNALING, HALLMARK_ANDROGEN_RESPONSE, HALLMARK_APOPTOSIS, HALLMARK_OXIDATIVE_PHOSPHORYLATION, and HALLMARK_PROTEIN_SECRETION, among others. Distinct pathway enrichment patterns were observed across PHRGclusters. Notably, HALLMARK_IL6_JAK_STAT3_SIGNALING and HALLMARK_HYPOXIA were enriched in PHRGcluster C1, whereas HALLMARK_DNA_REPAIR and related pathways were enriched in PHRGcluster C2. These results describe correlative differences in pathway activity across subtypes and do not establish causality (Fig. 4A). To examine associations between PHRGclusters and clinicopathological features, the distribution of clinical variables across clusters was visualized using ggplot. PHRGclusters C1 and C3 included a higher proportion of female patients compared with PHRGcluster C2, which exhibited worse prognosis (Fig. 4B). In the age ≤ 67 group, PHRGcluster C1 accounted for a higher proportion than PHRGcluster C2 (Fig. 4C). An alluvial (Sankey) diagram generated using ggalluvial showed that most patients in PHRGcluster C1 (favorable prognosis) were stage I–II and predominantly M0, whereas a substantial proportion of M1 cases were classified into PHRGcluster C2 (Fig. 4D).

### Construction of molecular subtypes and enrichment analysis based on pyroptosis- and hypoxia-related differentially expressed genes

**Fig. 5:**
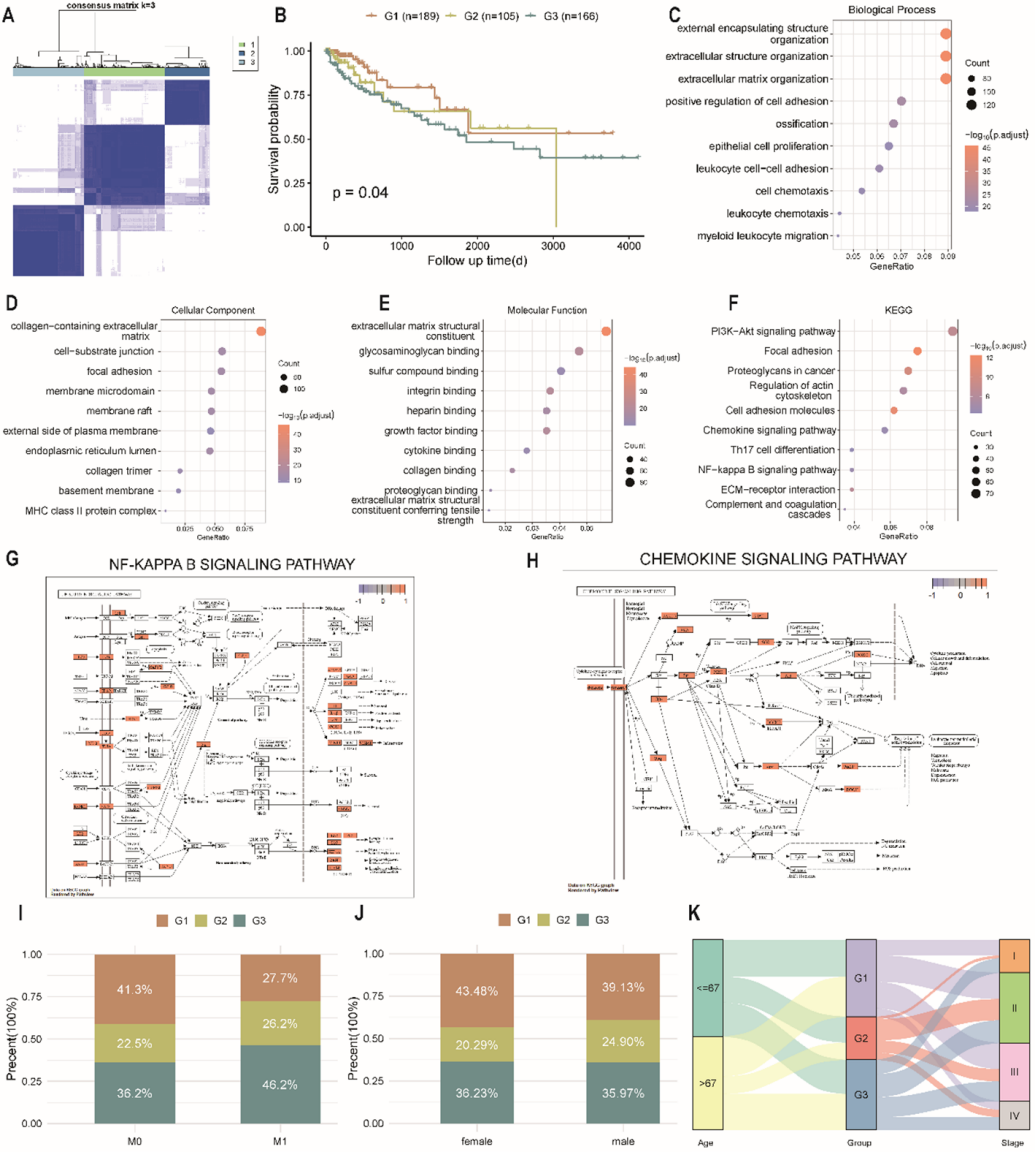
Construction of molecular subtypes and functional enrichment based on pyroptosis- and hypoxia-related differentially expressed genes (PHRDEGs). **A** Heatmap of the consensus matrix for PHRDEG-based clustering. **B** Kaplan–Meier survival curves of the three PHRDEGclusters. GO enrichment analysis of PHRDEGs in **C** biological process (BP), **D** cellular component (CC), and **E** molecular function (MF). **F** KEGG pathway enrichment analysis. **G** NF-κB signalling and **H** chemokine signalling pathway enrichment results. Distribution of PHRDEGclusters according to **I** TNM_M stage and **J** sex. **K** Alluvial diagram showing transitions among age, PHRDEGclusters, and tumour stage

To characterize biological differences across PHRGclusters, DEGs were identified using the limma package, yielding 1578 PHRDEGs (Table S5). Based on the PHRDEGs, an unsupervised consensus clustering analysis stratified patients into three PHRDEGclusters (Fig. 5A). Survival analysis revealed marked differences among the three PHRDEGclusters (Fig. 5B).

To explore functional characteristics of PHRDEGs, GO (Table 3) and KEGG (Table 4) enrichment analyses were performed. GO analysis indicated that PHRDEGs were enriched in extracellular matrix organization, extracellular structure organization and external encapsulating structure organization, positive regulation of cell adhesion, ossification, and leukocyte cell-cell adhesion within BP (Fig. 5C); collagen-containing extracellular matrix, endoplasmic reticulum lumen, cell-substrate junction, focal adhesion, membrane raft, and membrane microdomain within CC (Fig. 5D); and extracellular matrix structural constituent, integrin binding, glycosaminoglycan binding, growth factor binding, collagen binding, and heparin binding within MF (Fig. 5E). KEGG analysis showed enrichment in focal adhesion, rheumatoid arthritis, cell adhesion molecules, proteoglycans in cancer, leishmaniasis, Staphylococcus aureus infection, and the PI3K-Akt signalling pathway (Fig. 5F). The NF-κB and chemokine signalling pathways are presented in Fig. 5G and H, respectively.

**Table 3:** GO enrichment analysis results of differential genes of molecular subtypes of PHRGs by gene TCGA-CRC dataset

ONTOLOGY	ID	Description	*p*.adjust
BP	GO:0030198	Extracellular matrix organization	9.85E−47
BP	GO:0043062	Extracellular structure organization	9.85E−47
BP	GO:0045229	External encapsulating structure organization	1.28E−46
BP	GO:0045785	Positive regulation of cell adhesion	1.43E−23
BP	GO:0001503	Ossification	8.92E−23
BP	GO:0007159	Leukocyte cell-cell adhesion	1.61E−20
BP	GO:0060326	Cell chemotaxis	2.33E−19
BP	GO:0050673	Epithelial cell proliferation	5.93E−19
BP	GO:0097529	Myeloid leukocyte migration	7.94E−19
BP	GO:0030595	Leukocyte chemotaxis	1.22E−18
CC	GO:0062023	Collagen-containing extracellular matrix	6.34E−47
CC	GO:0005788	Endoplasmic reticulum lumen	1.72E−14
CC	GO:0030055	Cell-substrate junction	1.72E−14
CC	GO:0005925	Focal adhesion	1.72E−14
CC	GO:0045121	Membrane raft	1.73E−14
CC	GO:0098857	Membrane microdomain	1.73E−14
CC	GO:0005581	Collagen trimer	3.51E−12
CC	GO:0042613	MHC class II protein complex	1.62E−10
CC	GO:0005604	Basement membrane	1.14E−09
CC	GO:0009897	External side of plasma membrane	3.04E−09
MF	GO:0005201	Extracellular matrix structural constituent	4.93E−45
MF	GO:0005178	Integrin binding	2.76E−21
MF	GO:0005539	Glycosaminoglycan binding	2.76E−21
MF	GO:0019838	Growth factor binding	1.21E−20
MF	GO:0005518	Collagen binding	3.17E−17
MF	GO:0008201	Heparin binding	1.11E−16
MF	GO:0043394	Proteoglycan binding	2.02E−13
MF	GO:0019955	Cytokine binding	2.16E−12
MF	GO:1,901,681	Sulfur compound binding	4.03E−12
MF	GO:0030020	Extracellular matrix structural constituent conferring tensile strength	7.63E−11

*TCGA* The Cancer Genome Atlas, *CRC* Colorectal adenocarcinoma, *GO* Gene Ontology, *BP* Biological Process, *MF* Molecular Function, *CC* Cellular Component, *PHRGs* Pyroptosis And Hypoxia-Related Genes

**Table 4:** KEGG enrichment analysis results of differential genes of molecular subtypes of PHRGs by gene TCGA-CRC dataset

ID	Description	*p*.adjust
hsa04510	Focal adhesion	5.27E−13
hsa05323	Rheumatoid arthritis	3.43E−12
hsa04514	Cell adhesion molecules	4.62E−12
hsa05205	Proteoglycans in cancer	7.84E−11
hsa05140	Leishmaniasis	1.71E−10
hsa05150	Staphylococcus aureus infection	6.65E−10
hsa04151	PI3K–Akt signalling pathway	2.15E−09
hsa05144	Malaria	4.85E−09
hsa04512	ECM-receptor interaction	4.85E−09
hsa04640	Hematopoietic cell lineage	5.13E−09

*TCGA* The Cancer Genome Atlas, *CCR* Colorectal adenocarcinoma, *KEGG* Kyoto Encyclopedia of Genes and Genomes, *PHRGs* Pyroptosis And Hypoxia-Related Genes

To assess associations between PHRDEGclusters and clinicopathological characteristics, proportions of M stage and sex across clusters were compared using ggplot. In M0 disease, the proportion of PHRDEGcluster G1 (favorable prognosis) exceeded that of PHRDEGcluster G3 (unfavorable prognosis), whereas in M1 disease, PHRDEGcluster G3 accounted for a higher proportion than PHRDEGcluster G1 (Fig. 5I). Female patients also showed a higher proportion of PHRDEGcluster G1 than PHRDEGcluster G3 (Fig. 5J). The alluvial diagram indicated that patients aged ≤ 67 were more frequently classified into PHRDEGcluster G1, whereas most stage III–IV cases with unfavorable prognosis were classified into PHRDEGcluster G3 (Fig. 5K).

### Construction of pyroptosis- and hypoxia-related differentially expressed gene-related prognostic model and gene set enrichment analysis

**Fig. 6:**
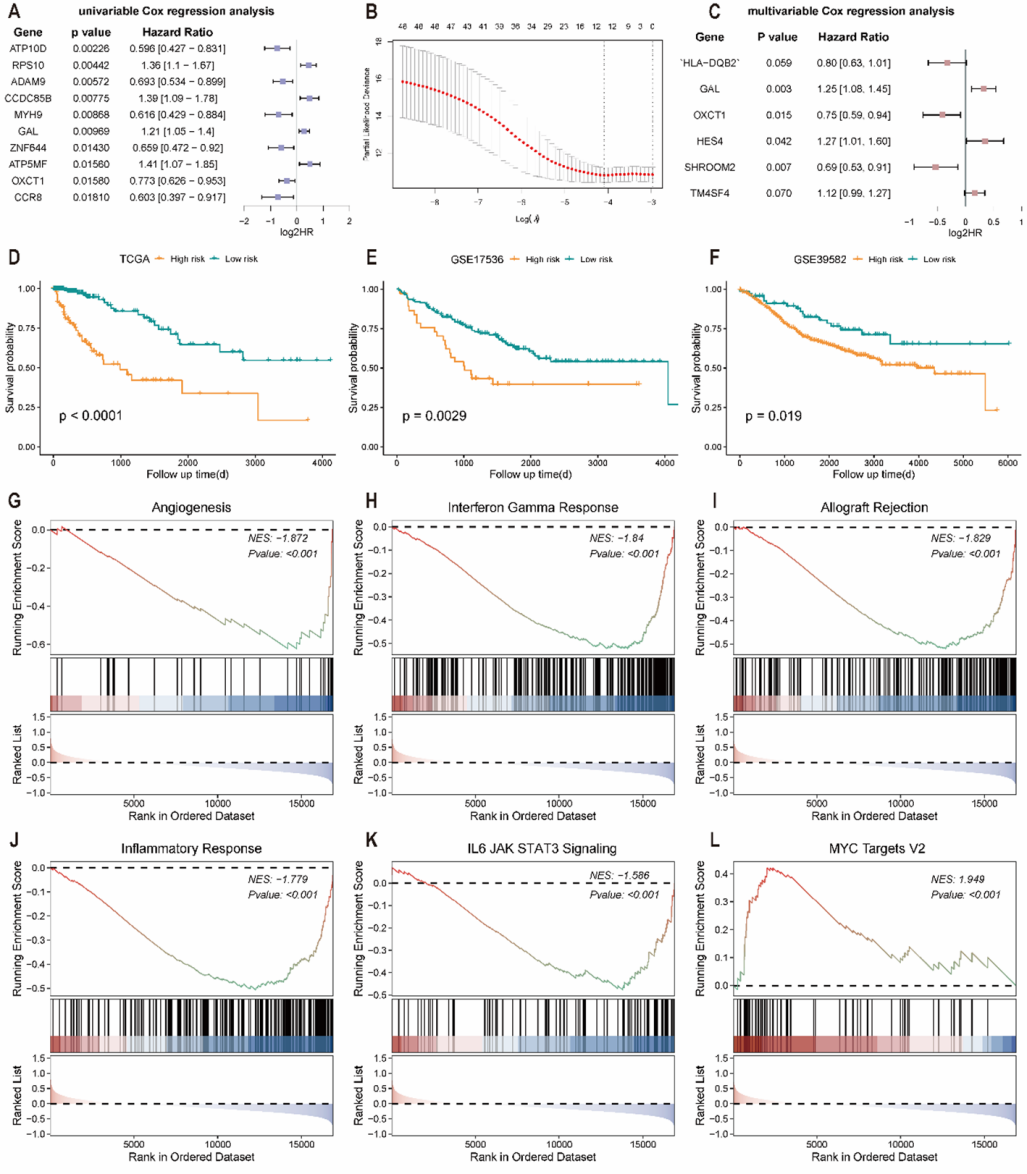
Construction and validation of the PHRDEG-related prognostic model and associated pathway enrichment. **A** Forest plot of univariate Cox regression analysis for PHRDEGs. **B** Cross-validation curve for least absolute shrinkage and selection operator (LASSO) regression. **C** Forest plot of multivariable Cox regression analysis identifying independent prognostic genes. Kaplan–Meier survival curves of different PHRDEGscore risk groups in the **D** TCGA, **E** GSE17536, and **F** GSE39582 cohorts. Gene set enrichment analysis (GSEA) of **G** angiogenesis, **H** interferon-gamma response, **I** allograft rejection, **J** inflammatory response, **K** IL6/JAK/STAT3 signalling, and **L** MYC targets V2 pathways

To quantify the prognostic impact of PHRDEGs at the individual level, a prognostic PHRDEGscore model was constructed. Univariate Cox regression analysis identified 48 genes with *P* < 0.05 for subsequent modeling (Fig. 6A, Table S6). LASSO regression was then applied to reduce collinearity, resulting in 13 candidate genes (Fig. 6B). Finally, multivariate Cox regression analysis established a six-gene prognostic model (Fig. 6C). The PHRDEGscore was calculated as:

$$\begin{aligned} {\mathrm{PHRDEGscore}} & =\left( {0.{\mathrm{23}} \times {\mathrm{GAL}}\;{\mathrm{expression}}} \right)+\left( {0.{\mathrm{24}} \times {\mathrm{HES4}}\;{\mathrm{expression}}} \right)+\left( {0.{\mathrm{12}} \times {\text{TM4SF4 expression}}} \right) \\ & \quad +\left( { - 0.{\mathrm{23}} \times {\mathrm{HLA}} - {\mathrm{DQB2}}\;{\mathrm{expression}}} \right)+\left( { - 0.{\mathrm{29}} \times {\mathrm{OXCT1}}\;{\mathrm{expression}}} \right)+\left( { - 0.{\mathrm{37}} \times {\mathrm{SHROOM2}}\;{\mathrm{expression}}} \right). \\ \end{aligned} $$

Patients in the TCGA-CRC cohort were stratified into high- and low-PHRDEGscore groups using the optimal cutoff value. Kaplan–Meier analysis showed that patients with a low PHRDEGscore had significantly longer survival than those with a high PHRDEGscore (Fig. 6D). External validation in GSE17536 (Fig. 6E) and GSE39582 (Fig. 6F) further supported the prognostic robustness of the PHRDEGscore.

To investigate biological differences between PHRDEGscore groups, GSEA was performed (Table 5). Angiogenesis (Fig. 6G), Interferon Gamma Response (Fig. 6H), Allograft Rejection (Fig. 6I), Inflammatory Response (Fig. 6J), and IL6/JAK/STAT3 signalling (Fig. 6K) were enriched in the low-PHRDEGscore group, whereas MYC Targets V2 (Fig. 6L) was enriched in the high-PHRDEGscore group.

**Table 5:** Results of GSEA enrichment analysis for high and low risk of risk score based on TCGA-CRC dataset

ID	NES	*p*.adjust
HALLMARK_ALLOGRAFT_REJECTION	− 1.828995683	8.60E−10
HALLMARK_EPITHELIAL_MESENCHYMAL_TRANSITION	− 2.17185729	8.60E−10
HALLMARK_INFLAMMATORY_RESPONSE	− 1.778902735	8.60E−10
HALLMARK_INTERFERON_GAMMA_RESPONSE	− 1.839725665	8.60E−10
HALLMARK_UV_RESPONSE_DN	− 1.967641795	8.60E−10
HALLMARK_KRAS_SIGNALING_UP	− 1.762044317	1.82E−09
HALLMARK_TNFA_SIGNALING_VIA_NFKB	− 1.667103463	1.41E−07
HALLMARK_MITOTIC_SPINDLE	− 1.619051544	3.96E−06
HALLMARK_COMPLEMENT	− 1.585373817	1.32E−05
HALLMARK_IL2_STAT5_SIGNALING	− 1.568917678	2.19E−05

*TCGA* The Cancer Genome Atlas, *CRC* Colorectal adenocarcinoma, *GSEA* Gene-Set Enrichment Analysis

### Analysis of immune cell infiltration

**Fig. 7:**
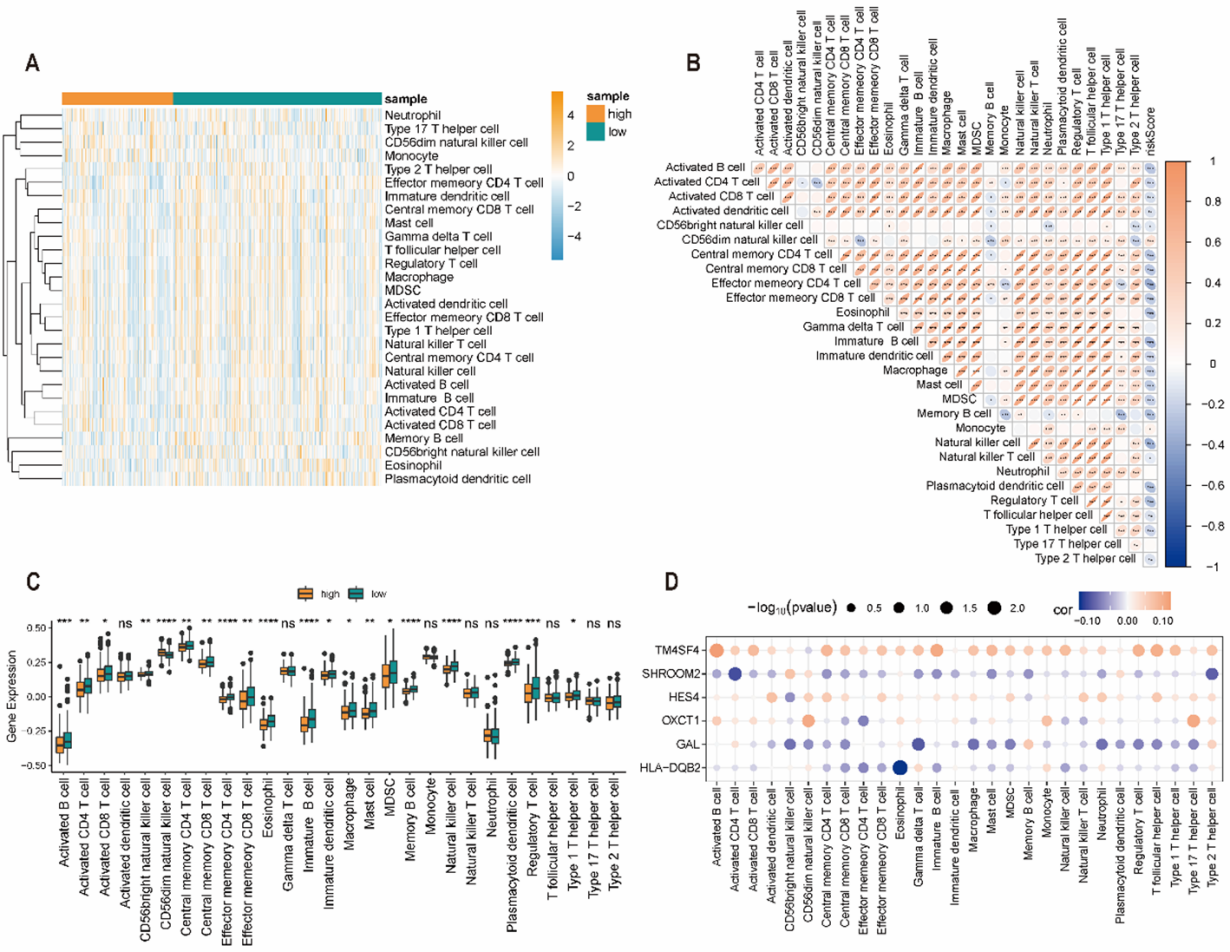
Immune landscape associated with the PHRDEGscore. **A** Heatmap showing immune cell infiltration levels across different PHRDEGscore groups. **B** Correlation matrix among infiltrating immune cell types. **C** Differences in immune cell infiltration between high- and low-PHRDEGscore groups. **D** Correlations between immune cell infiltration and expression of prognostic model genes

To examine the association between the PHRDEGscore and the immune microenvironment, ssGSEA was used to estimate infiltration levels of each immune cell subset. Neutrophils and type 17 T helper cells showed higher infiltration in the high-PHRDEGscore group, whereas central memory CD4+ T cells, natural killer cells, and type 1 T helper cells were more enriched in the low-PHRDEGscore group (Fig. 7A, Table S7). Correlation analysis among immune cell subsets indicated strong positive correlations between activated dendritic cells and myeloid-derived suppressor cells. CD56dim natural killer cells were negatively correlated with effector memory CD4+ T cells, and the PHRDEGscore was negatively correlated with effector memory CD4+ T cells (Fig. 7B). In addition, infiltration levels of more than 70% of immune cell subsets differed significantly between PHRDEGscore groups, with most immune cell subsets showing higher infiltration in the low-PHRDEGscore group (Fig. 7C). At the gene level, TM4SF4 was positively correlated with most immune cell subsets, whereas HLA-DQB2 and GAL were negatively correlated with most immune cell subsets (Fig. 7D).

### Association with immunotherapy outcomes

**Fig. 8:**
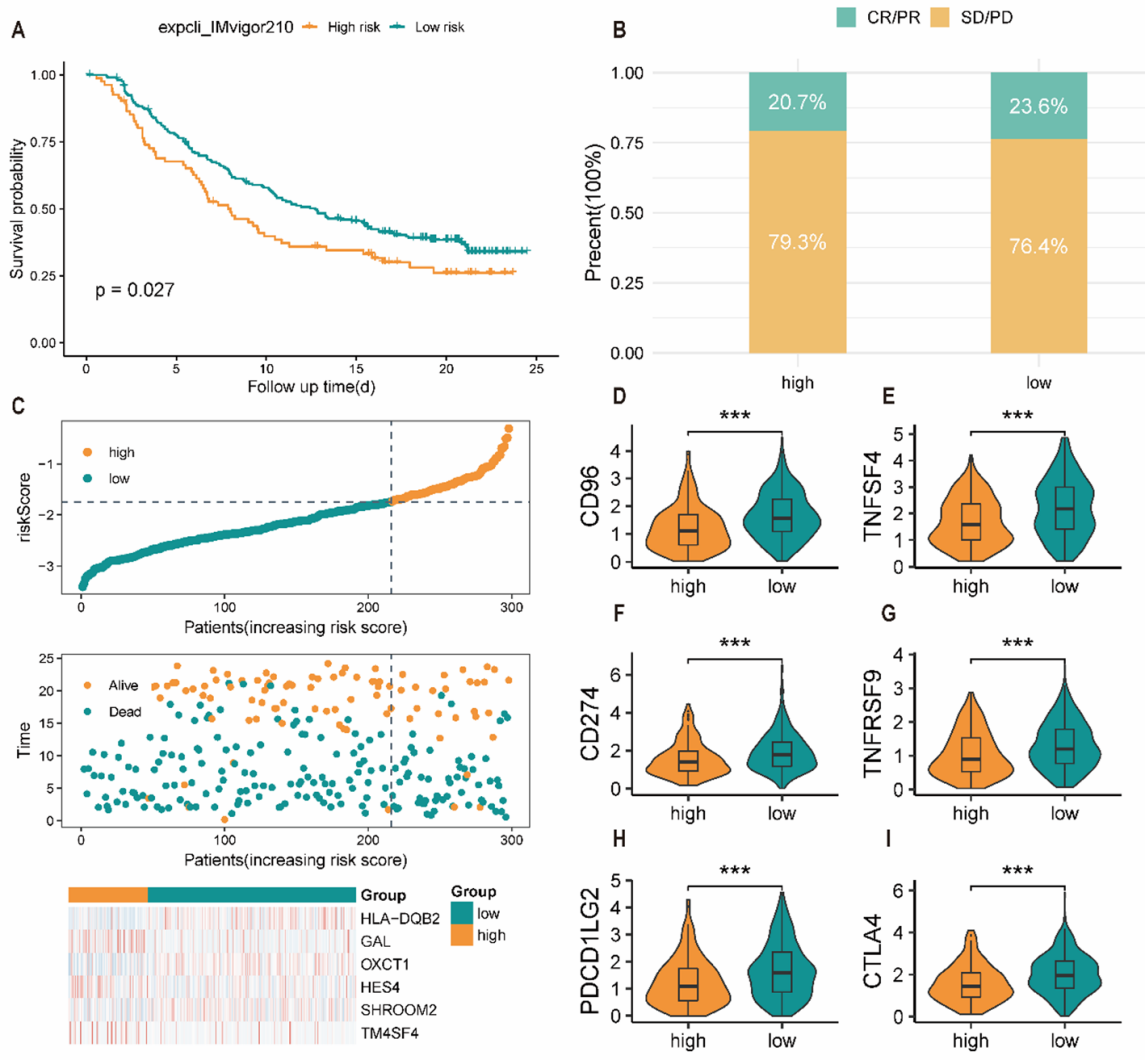
Exploratory analysis of the PHRDEGscore and immunotherapy response in a non-CRC cohort. **A** Kaplan–Meier survival curves of different PHRDEGscore groups in the IMvigor210 immunotherapy-treated cohort. **B** Comparison of immunotherapy response rates between PHRDEGscore groups. **C** Distribution of PHRDEGscore, survival status, and expression patterns of prognostic genes. Expression levels of immune checkpoint genes **D** CD96, **E** TNFSF4, **F** CD274, **G** TNFRSF9, **H** PDCD1LG2, and **I** CTLA4 in different PHRDEGscore groups

To explore a potential link between the PHRDEGscore and immunotherapy outcomes, we leveraged the IMvigor210 cohort, a publicly available dataset of bladder cancer patients treated with immunotherapy, as an exploratory external reference. This was necessary due to the current lack of publicly available CRC immunotherapy cohorts with complete clinical and genomic annotation. In this non-CRC cohort, patients with a low PHRDEGscore showed longer survival (Fig. 8A). The proportion of immunotherapy responses was higher in the low-PHRDEGscore group (Fig. 8B). Consistently, patients in the high-PHRDEGscore group showed a higher likelihood of death (Fig. 8C). These findings suggest a potential, though indirect, association. Immune checkpoints are key regulatory mechanisms involved in immune tolerance. To assess the relationship between the PHRDEGscore and immune checkpoint expression, immune checkpoint genes were compared between PHRDEGscore groups. Most immune checkpoint genes were differentially expressed between groups, with higher expression levels observed in the low-PHRDEGscore group (Fig. 8D–I). These observations are hypothesis-generating and do not imply that the signature can predict or guide clinical immunotherapy in CRC.

### Construction of a nomogram prediction model

**Fig. 9:**
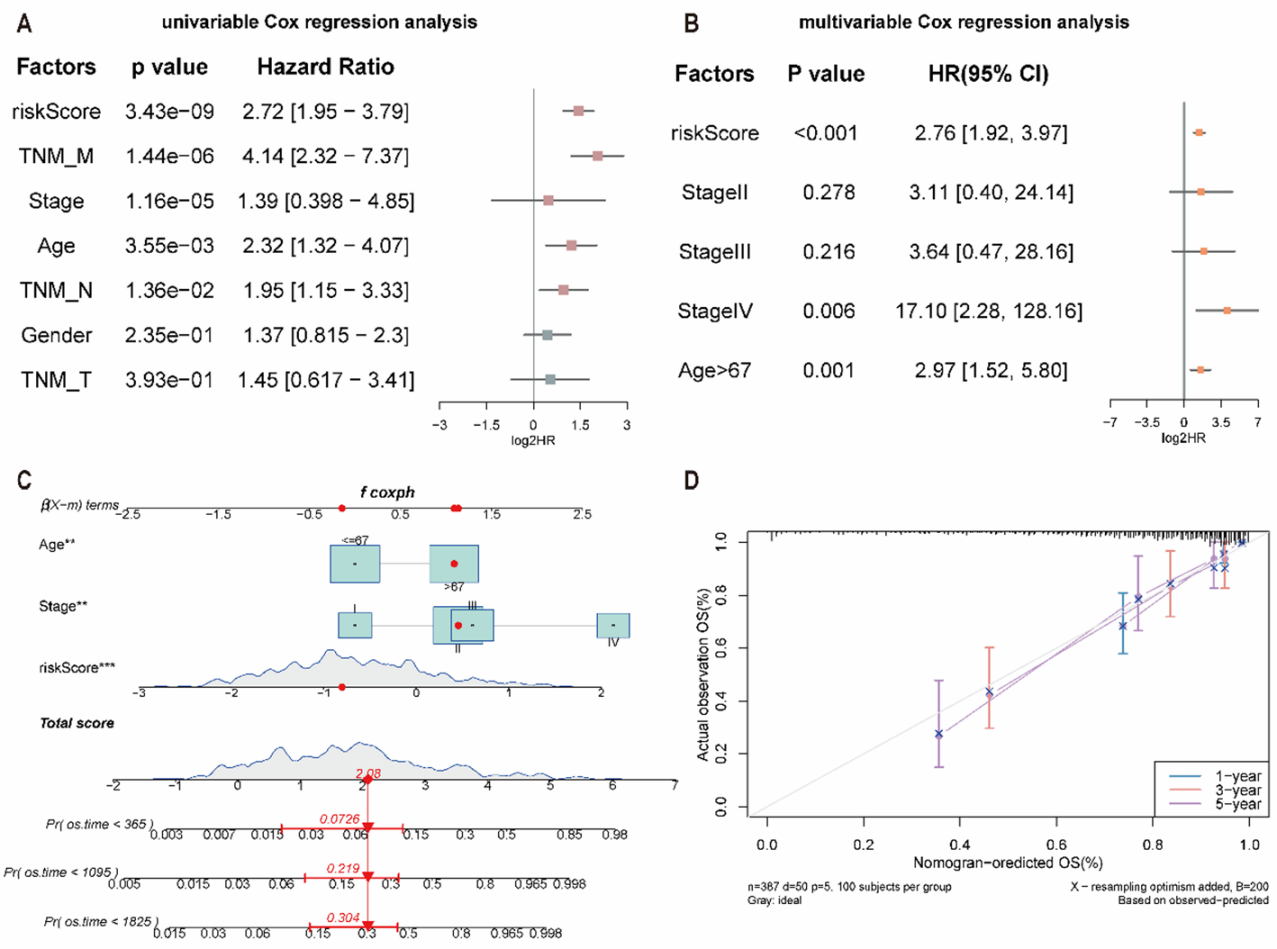
Development of a nomogram model for survival prediction in colorectal cancer (CRC). Forest plots of **A** univariate and **B** multivariate Cox regression analyses incorporating clinical variables and the PHRDEGscore. **C** Nomogram integrating independent prognostic factors to predict survival probability. **D** Calibration curve evaluating the agreement between predicted and observed survival

We next evaluated whether the PHRDEGscore served as an independent prognostic factor in CRC. Univariate Cox regression analysis showed that the PHRDEGscore, M stage, TNM stage, age, and N stage were significantly associated with overall survival (Fig. 9A; Table 6). Multivariate Cox regression analysis indicated that the PHRDEGscore, TNM stage, and age remained significantly associated with CRC prognosis (Fig. 9B; Table 7). Based on these results, a nomogram incorporating the PHRDEGscore, TNM stage, and age was constructed to predict survival probability (Fig. 9C). Calibration curves demonstrated good agreement between nomogram-predicted and observed survival probabilities (Fig. 9D).

**Table 6:** Univariate cox regression results of pyroptosis and hypoxia gene-related score on clinical prognosis

	Hazard Ratio (HR)	lower 95%CI	upper 95%CI	*p* value
riskScore	2.72	1.95	3.79	3.43E−09
TNM_M	4.14	2.32	7.37	1.44E−06
Stage	1.39	0.398	4.85	1.16E−05
Age	2.32	1.32	4.07	0.00355
TNM_N	1.95	1.15	3.33	0.0136
Gender	1.37	0.815	2.3	0.235
TNM_T	1.45	0.617	3.41	0.393

**Table 7:** Multivariate cox regression results of pyroptosis and hypoxia gene-related score on clinical prognosis

	Hazard Ratio (HR)	lower 95%CI	upper 95%CI	*p* value
riskScore	2.76	1.92	3.97	< 0.001
StageII	3.11	0.4	24.14	0.278
StageIII	3.64	0.47	28.16	0.216
StageIV	17.1	2.28	128.16	0.006
Age > 67	2.97	1.52	5.8	0.001

### In-vitro verification of signature genes by qRT-PCR

**Fig. 10:**
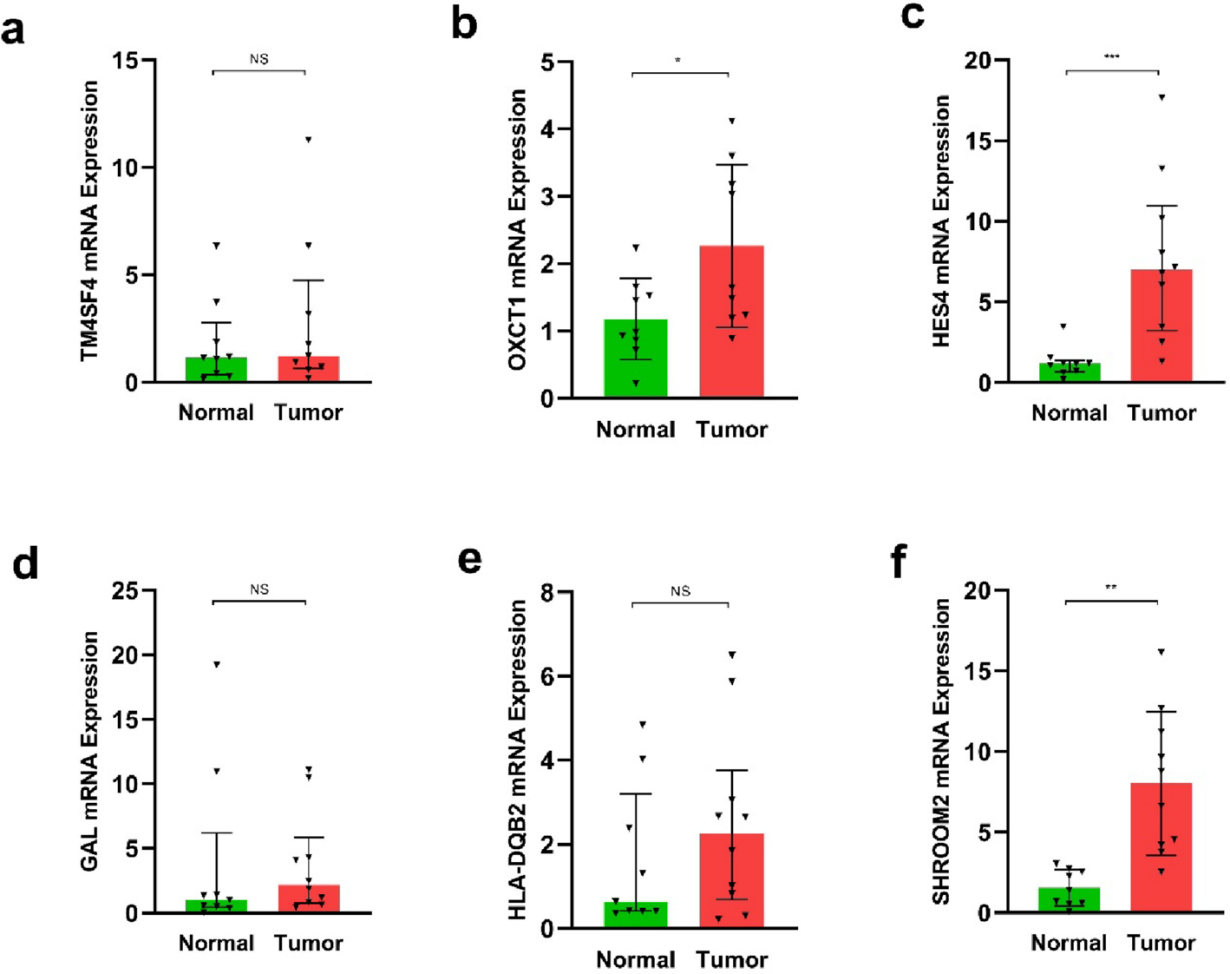
qRT-PCR validation of prognostic gene expression in CRC tissues. Relative mRNA expression levels of **a** TM4SF4, **b** OXCT1, **c** HES4, **d** GAL, **e** HLA-DQB2, and **f** SHROOM2 in 10 paired CRC tissues and adjacent non-cancerous tissues. Statistical significance is indicated as *p < 0.05; **p < 0.01; ***p < 0.001; NS, not significant

qRT-PCR was performed to assess expression of the six signature genes in 10 paired CRC and adjacent normal tissues collected after surgical resection at The First People’s Hospital of Jiujiang City. Among the six genes, HES4, SHROOM2, and OXCT1 showed significant expression differences between tumour and adjacent normal tissues, whereas TM4SF4, GAL, and HLA-DQB2 did not show statistically significant differences (Fig. 10). These results indicate that HES4, SHROOM2, and OXCT1 exhibit consistent differential expression in this small paired cohort, providing preliminary expression-level support for their inclusion in the prognostic signature.

### Western blotting

**Fig. 11:**
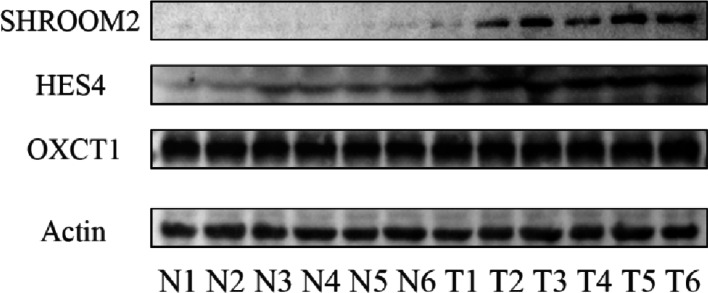
Western blot validation of selected prognostic genes in CRC. Representative western blot images showing increased protein expression of HES4 and SHROOM2 in CRC tissues compared with paired adjacent normal tissues (*n* = 6 pairs), whereas OXCT1 did not show a significant difference. Actin was used as a loading control

Western blotting was performed to examine protein expression of HES4, SHROOM2, and OXCT1 in six paired CRC and adjacent normal tissues. HES4 and SHROOM2 protein levels were significantly increased in CRC tissues, whereas OXCT1 did not show a significant difference between tumour and adjacent normal tissues (Fig. 11).

## Discussion

Malignant tumour is highly heterogeneous regardless of whether they originate from the same or different sites, and their characteristics are related to the tumour microenvironment [15]. The tumour microenvironment is a complex system that contains tumour, immune, and stromal cells, as well as the extracellular matrix, and is associated with the survival and invasion of tumour cells [45]. The development of effective anticancer treatments is challenged by the complex tumour microenvironment. Owing to the rapid and uncontrolled proliferation of tumour cells, which accelerates oxygen consumption, hypoxia is a classic characteristic of the tumour microenvironment in almost all solid tumour [46]. Pyroptosis is a proinflammatory programmed cell death. Studies have shown that proinflammatory pyroptosis affects the tumour immune microenvironment and antitumour immune effects [47, 48]. In this study, we constructed pyroptosis- and hypoxia-related molecular characteristics and a prognostic signature for CRC, providing an additional framework for describing tumour heterogeneity and its potential therapeutic implications.

The expression of most PHRGs differed markedly between colorectal tumour and normal tissues. Consensus clustering stratified CRC samples into four PHRGclusters, which exhibited distinct prognoses and biological behaviours. We identified 1578 PHRDEGs among these clusters. GO and KEGG analyses showed that the PHRDEGs were enriched in extracellular matrix–related processes and immune-associated pathways, supporting an association between these gene programs and CRC biology rather than demonstrating direct mechanistic causality. Composition of the extracellular matrix is associated with the characteristics and prognosis of malignant tumour [49]. The extracellular matrix consists of collagen, elastin, fibronectin, hyaluronic acid, proteoglycan, and glycoproteins that provide structural support by surrounding cells [50]. Hypoxia and amplified inflammatory reactions within the tumour microenvironment can alter extracellular matrix composition, promote angiogenesis, provide sustained proliferative signals, facilitate resistance to cell death, and ultimately contribute to invasion and metastasis [51–53].

Given tumour heterogeneity, we used LASSO and Cox regression analyses to develop a prognostic PHRDEGscore signature derived from PHRDEGs in the TCGA-CRC dataset and further supported its robustness in two external datasets, GSE17536 and GSE39582. The prognostic model comprised six genes: HLA-DQB2, GAL, OXCT1, HES4, SHROOM2, and TM4SF4. This signature likely reflects the transcriptional consequences of a complex interplay between hypoxia, inflammation, and immune remodeling, but our study does not establish a causal mechanistic framework. To contextualize the biological plausibility of these genes, we summarized their previously reported functions and knowledge gaps (Table 8). For instance, genes such as HES4 and OXCT1 have established links to hypoxia and metabolic adaptation [54, 55], while HLA-DQB2 is a core component of antigen presentation machinery [56]. Other genes like SHROOM2 and TM4SF4 are involved in cell structure and metastasis [57, 58], and GAL is a neuropeptide with emerging roles in cancer [59]. The summary in Table 8 underscores that while individual genes have relevant functions, their collective role in a coordinated hypoxia-pyroptosis axis in CRC is speculative and requires experimental validation.

**Table 8:** Summary of prior evidence for each signature gene

Gene	Reported function (brief)	Evidence in CRC or related cancers	Hypoxia association	Immune/pyroptosis relevance	Key knowledge gaps
HES4	Notch pathway transcriptional target; regulates cell differentiation and epithelial-mesenchymal transition	Aberrantly expressed in CRC; BEST4 relays HES4 to suppress EMT in CRC cells [60]; associated with tumor progression and metastasis in osteosarcoma [61]	Hypoxia-mediated HES4 promotes proliferation and motility in hepatocellular carcinoma [55]; enriched in hypoxic tumor microenvironments	Notch signaling implicated in immune modulation and tumor-immune interactions; potential link to immunotherapy sensitivity [62]	Direct functional role in CRC hypoxia-pyroptosis axis unproven; lack of mechanistic studies in CRC-specific hypoxic conditions; causal link to pyroptosis unclear
SHROOM2	Cytoskeletal regulator via RhoA-ROCK pathway; mediates cell motility and actin organization	X-linked CRC susceptibility locus [63]; inhibits tumor metastasis in nasopharyngeal carcinoma [57]; higher expression associated with favorable survival in multiple cancers	Indirect: cytoskeletal remodeling may respond to microenvironmental stress, but direct hypoxia link not established	Cytoskeletal organization influences immune cell trafficking and stromal remodeling; potential role in shaping immune microenvironment [64]	No direct functional validation in CRC; hypoxia-SHROOM2 interaction unknown; mechanism linking SHROOM2 to pyroptosis or immune infiltration not established
OXCT1	Rate-limiting enzyme in ketone body metabolism; catalyzes ketone body utilization	Metabolic tumor suppressor inhibiting CRC liver metastasis via metabolic-epigenetic-Wnt axis [65]; context-dependent roles in different cancers [66]	Metabolic adaptation under microenvironmental stress including hypoxia; ketone metabolism linked to tumor metabolic reprogramming [67]	OXCT1 in tumor-associated macrophages reprograms immune microenvironment [68]; potential indirect link to inflammatory signaling	Role in CRC-specific hypoxia-pyroptosis crosstalk unclear; conflicting reports on pro- vs. anti-tumor roles across cancer types; functional validation in CRC lacking
GAL	Neuropeptide galanin; involved in neuroimmune and inflammatory signaling	Upregulated in CRC liver metastasis; associated with altered glucose metabolism and poor prognosis in stage II CRC [69]; elevated in serum and tissue of CRC patients [70]	Unknown: direct hypoxia-GAL link not reported	May intersect with neuroimmune and inflammatory pathways; galanin receptors implicated in CRC progression [71]	Mechanistic link to hypoxia and pyroptosis unestablished; functional role in immune modulation unclear; causal relationship with CRC prognosis requires validation
TM4SF4	Tetraspanin L6 domain family member; regulates cell adhesion, migration, and signaling platforms	Promotes CRC metastasis via EMT and stem cell marker upregulation [72]; overexpressed in hepatocellular carcinoma and linked to metastasis [73]	Unknown: no direct evidence linking TM4SF4 to hypoxia response	Potential intersection with immune-regulatory signals including PD-L1-related pathways; tetraspanins implicated in tumor-immune interactions	Direct role in hypoxia-pyroptosis axis unproven; mechanism linking TM4SF4 to immune infiltration unclear; functional validation in CRC hypoxic conditions lacking
HLA-DQB2	MHC class II molecule; mediates antigen presentation to CD4+ T cells	Higher expression associated with better overall survival in breast cancer and other malignancies [56]; HLA class II expression linked to favorable prognosis and immunotherapy response [74]	Unknown: no direct hypoxia-HLA-DQB2 link reported	Central to adaptive immune engagement and antitumor immunity; MHC class II expression correlates with immune infiltration and checkpoint inhibitor response	Specific role of HLA-DQB2 in CRC not well-characterized; relationship to hypoxia-induced immune suppression unclear; functional link to pyroptosis-driven inflammation unknown

This table summarizes published evidence supporting the biological plausibility of the six-gene signature. However, the mechanistic integration of these genes into a unified hypoxia-pyroptosis-immune axis in CRC remains largely unexplored and requires experimental validation

The tumour microenvironment is the internal environment for production and survival of tumour cells and is a complex system [45]. We found that the PHRDEG-related prognostic signature was associated with characteristics of the immune microenvironment. The majority of immune cells, such as activated CD8+ T and activated B cells, were highly infiltrated in the low PHRDEGscore group. CD8+ T cells have vital functions in antitumour immunity because of their killing effect. High infiltration of CD8+ T cells is believed to be related to a better prognosis for malignant tumours [75]. Recently, immunotherapy has achieved remarkable success in the treatment of many solid malignancies, including melanoma, hepatocellular cancer, endometrial cancer, and squamous cell carcinoma of the head and neck [76–79]. However, not all patients benefit from immunotherapy because of the complex tumour immune microenvironment. Immune checkpoint therapy is an immunotherapy modality approved in 2017 to treat patients with CRC that have defects in mismatch repair or severe mutations with a high degree of microsatellite instability [80].

### Limitations

This study has several significant limitations that must be acknowledged. First, the signature and molecular patterns were derived from retrospective public datasets; therefore, the findings should be interpreted as associative and hypothesis-generating rather than causal. Second, our experimental validation was preliminary. The sample size was small (*n* = 10 for qRT-PCR, *n* = 6 for western blotting) and lacked stratification by clinical stage or molecular subtype, which limits the generalizability of the findings. Crucially, these experiments only confirmed expression-level differences and did not provide functional evidence. Future work is essential to elucidate the mechanistic roles of these signature genes, which would require knockdown or overexpression experiments in CRC cell lines, ideally under hypoxic conditions, to assess their impact on pyroptosis, invasion, and immune marker expression. Third, the analysis of immunotherapy response was exploratory and has major caveats. The association was observed in a non-CRC cohort (IMvigor210 bladder cancer), and therefore the results cannot be directly extrapolated to CRC. Furthermore, the analysis was not adjusted for known predictive biomarkers of immunotherapy response, such as microsatellite instability (MSI) status or tumour mutational burden (TMB). The conclusions drawn from this analysis are therefore strictly hypothesis-generating and underscore the need for validation in CRC-specific immunotherapy cohorts with comprehensive biomarker annotation. Finally, prospective validation is required. The prognostic value of the PHRDEGscore must be confirmed in independent, well-annotated prospective clinical cohorts before it can be considered for any clinical application.

## Conclusion

In summary, we used PHRGs to establish molecular characteristics and a prognostic signature for CRC using public datasets. This work highlights hypoxia- and pyroptosis-related molecular patterns that have an associative relationship with prognosis and immune features in CRC. However, these findings are exploratory and hypothesis-generating. The signature’s clinical relevance is currently unproven, as it requires rigorous validation through prospective studies in independent, well-annotated clinical cohorts and functional experiments before any consideration for clinical translation.

## Supplementary Information

Below is the link to the electronic supplementary material.

Supplementary Material 1.

Supplementary Material 2.

Supplementary Material 3.

Supplementary Material 4.

Supplementary Material 5.

Supplementary Material 6.

Supplementary Material 7.

## Data Availability

The original contributions presented in the study are included in the article. Further inquiries can be directed to the corresponding author.
